# Bacterial biofilms in calcium oxalate kidney stones: Where do we stand?

**DOI:** 10.1073/pnas.2606646123

**Published:** 2026-06-09

**Authors:** Carlos González González, Federico Zorzi, Michel Daudon, Olivier Traxer

**Affiliations:** ^a^ Endolase Lab, Groupe de Recherche Clinique n°20–Sorbonne Université, Procédés et Ingénierie en Mécanique et Matériaux Lab Arts et Métiers ParisTech, Paris 75020, France; ^b^ Service d’Urologie, Assistance Publique–Hôpitaux de Paris, Hôpital Tenon, Sorbonne Université, Paris 75020, France; ^c^ Procédés et Ingénierie en Mécanique et Matériaux, UMR 8006 CNRS–Arts et Métiers ParisTech, Paris 75013, France; ^d^ INSERM, UMR S 1155, Physiology Unit, Hôpital Tenon, Paris 75020, France

We have read the structural evidence provided by Schmidt et al. (1), who describe how bacterial biofilms and extracellular DNA (eDNA) are intercalated within calcium-based kidney stones. Using multimodal imaging approaches—including high-resolution microscopy and molecular characterization—they demonstrate that biofilm-derived organic material is embedded within lamellar mineral growth zones and integrated throughout the stone matrix. The study challenges the traditional view of calcium stone formation as a predominantly abiotic physicochemical process, suggesting microbial components may participate in mineral accretion. However, further clarification of how these findings relate to established nucleation pathways would strengthen interpretation of biofilm material as an intrinsic contributor and help define its position within recognized hierarchies of stone initiation.

First, the study reports biofilm presence in stones from patients without documented urinary tract infection, challenging the concept of a strictly abiotic process in calcium oxalate stone formation. Contemporary evidence demonstrates that urine is not sterile, even in asymptomatic individuals (2). More detailed documentation of preoperative midstream and renal pelvic urine cultures prior to stone retrieval would help determine whether observed biofilm structures reflect concurrent colonization, historical incorporation during stone growth, or other temporal dynamics. Structural heterogeneity during nucleation can allow transitions in mineral composition over time, reflecting dynamic physicochemical and microenvironmental conditions, as illustrated by calcium-phosphate stone formation (3).

Second, calcium-based stone nucleation occurs through distinct structural mechanisms, including overgrowth on Randall’s plaque and intratubular plugging, substrates spatially and temporally linked to stone initiation (4). The demonstration of biofilm material within lamellar growth zones supports its integration during development and suggests a role in amplifying heterogeneous nucleation. However, without explicit localization to the earliest mineral nidus—particularly in papillary stones exhibiting plaque attachment—the data may remain compatible with biofilms acting in a contributory capacity alongside established abiotic pathways rather than demonstrating them as primary initiating substrates.

Third, stone morphology and internal architecture encode dominant pathogenic pathways and correlate with specific metabolic phenotypes and recurrence risks (5). If biofilm incorporation contributes broadly to calcium stone formation, its prevalence and spatial distribution may vary across defined morphologic phenotypes, including calcium oxalate subtypes and carbapatite variants with distinct etiologies. Stratified analysis across established morphologic categories could clarify whether biofilm intercalation represents a generalized initiating process or a context-dependent contributor.

Finally, prospective data from our center illustrates the complexity of bacterial dynamics in stone disease. In a cohort of more than 200 consecutive flexible ureteroscopies with systematic intraoperative cultures, de novo renal pelvic culture positivity occurred in 7.1% of patients despite negative preoperative bladder cultures. Importantly, positivity detected before endourological treatment was the strongest predictor of postoperative infectious complications. These findings suggest that microbial presence may be spatially and temporally heterogeneous within the upper urinary tract, potentially confined to intrarenal microenvironments not captured by routine sampling.

Correlating stone microarchitecture with intraoperative renal pelvic cultures and clinical outcomes could help determine whether biofilm material reflects active microbial niches with pathophysiological relevance or residual incorporation within mineral matrices. Integrating multimodal imaging, morphologic phenotyping, and staged microbiologic sampling may therefore clarify the dynamics and clinical implications of biofilm intercalation in calcium stone formation.
